# 

**Published:** 1824-09

**Authors:** 


### MONTHLY CATALOGUE OF MEDICAL BOOKS.

*** *It having been hinted to us that gentlemen, sending copies of their Works, prefer having their titles given at length, it is our intention, in future, to comply with this suggestion: but it is to be observed, that no books can be entered on this List except those sent to us for the purpose;—finding, in the lists hitherto transmitted, that the names of works have frequently been given as published, which have not appeared for weeks, or even months after.*

Medical and Surgical Cases; selected during a Practice of thirty-eight Years. By EDWARD SUTLIFFE, Queen-street, London.—Pp. 628. London, 1824.

Remarks on an Institution for the Preparation and Use of Artificial Mineral Waters, in Great Britain. By Dr. STRUVE, of Dresden, Member of the Societies of Mineralogy in Dresden, and of Natural History in Leipzig, &c. &c. To which are added, Testimonials, by Dr. KREYSIG, of Dresden, and Dr. CLARUS, of Leipzig.—Pp. 16. London, 1824.

System of Anatomical Plates, &c. &c. By JOHN LIZARS, F.R.S.E. Part V. Muscles and Joints of the Upper and Lower Extremities.—Edinburgh, 1824.

Principles of Medical Science and Practice. Part I. Physiology. By HARDWICKE SHUTE, M.D. Physician to the General Infirmary, and to the County and City Lunatic Asylum, Gloucester.—London, 1824.

Original Cases, with Dissections and Observations, illustrating the Use of the Stethoscope and Percussion in the Diagnosis of the Diseases of the Chest; also Commentaries on the same Subjects, selected and translated from AVENBRUGGER, CORVISART, LAENNEC, and others. By JOHN FORBES, M.D. Physician to the Chichester Dispensary.—London, 1824.

De Medulla Spinali nervisque ex ed proderentibus Annotationes Anatomico-physiologicæ. Auctore CAROLO FRANCISCO BELLENGERI, Regiæ Scientiarum Academiæ et Collegii Medici Taurinensis Membro; Imp. et Reg. Scientiarum, Litterarum, et Artium Academie, Patavinæ; Sodali Regiæ Domus Medico Augustæ, Taurinorum ex Typographia Regiæ.—1823.

Analyse des Travaux de l'Academie Royale des Sciences, pendant l'année 1823. Partie Physique. Par M. le Baron CUVIER, Secretaire perpetuel.

Histoire Medicale de la Fievre Jaune, observée en Espagne, et particulièrement en Catalogue, dans l'année 1821. Par BALLY, FRANCOIS, et PARISET.——A Paris, 1823.

Leçons sur les Epidemies, et l'Hygiene publique. Par F. EMP. FODERE, faites a la Faculté de Medecine de Strasburg. Tome IV.—A Paris, 1824.

Observations illustrative of the Nature and Treatment of the prevailing Disorders of the Stomach and Liver. By JOHN THOMAS GRAHAM, M.D. Member of the Royal College of Surgeons.—London, 1824.

Concise Narrative of an Ophthalmia which prevailed in a Detachment of his Majesty's 44th Regiment, on their Voyage to Calcutta, in the Summer of 1822. Together with an Account of other Tropical Diseases, and their Treatment, on Board the H. C. Ship Warren Hastings. By RICHARD JONES, Leamington, Fellow of the Royal College of Surgeons, and late Assistant Surgeon to the Warren Hastings, &c.—Warwick, 1824.

Du Froid, et de son Application dans les Maladies; Considerations Physiologiques et Therapeutiques; Observations, Corollaires. Par S. TANCHOU, Docteur en Medecine de la Faculté de Paris; Membre de la Legion d'Honneur, de la Societé Medicale d'Emulation, &c. &c.—Paris, 1824.

A Practical Treatise on Diseases of the Skin; comprehending an Account of such Facts as have been recorded on these Subjects, with original Observations. The whole arranged with a view to illustrate the Constitutional Causes of these Diseases, as well as their local Character. By SAMUEL PLUMBE, Member of the Royal College of Surgeons, of the Medico-Chirurgical Society, &c. &c.—London, 1824.

### NOTICE TO CORRESPONDENTS.

*Communications have been received from* Dr. BALFOUR, Mr. WHITE, *&c. &c. to which we hope to give insertion in our ensuing Number.*

